# Proteomic analysis of Malaysian Horseshoe crab (*Tachypleus gigas*) hemocytes gives insights into its innate immunity host defence system and other biological processes

**DOI:** 10.1371/journal.pone.0272799

**Published:** 2022-08-10

**Authors:** Ismail Abiola Adebayo, Mohd Afiq Hazlami Habib, Maria E. Sarmiento, Armando Acosta, Nik Soriani Yaacob, Mohd Nazri Ismail

**Affiliations:** 1 Faculty of Biomedical Sciences, Department of Microbiology and Immunology, Kampala International University, Ishaka, Bushenyi, Uganda; 2 Analytical Biochemistry Research Centre, Universiti Sains Malaysia, Bayan Lepas, Pulau Pinang, Malaysia; 3 Department of Clinical Biology, School of Medicine and Pharmacy, College of Medicine and Health Sciences, University of Rwanda, Kigali, Rwanda; 4 School of Health Sciences, Universiti Sains Malaysia, Kelantan, Malaysia; 5 Department of Chemical Pathology, School of Medical Sciences, Universiti Sains Malaysia (USM), Kelantan, Malaysia; 6 Institute for Research in Molecular Medicine, Universiti Sains Malaysia, USM, Pulau Pinang, Malaysia; Hospital for Sick Children, CANADA

## Abstract

Horseshoe crabs are one of the most studied invertebrates due to their remarkable innate immunity mechanism and biological processes. In this work, the proteins of the lipopolysaccharides (LPS)-stimulated and non-stimulated hemocytes of Malaysian *Tachypleus gigas* were profiled using LC-MS/MS. A total of 154 proteins were identified in both types of samples. Additionally, seventy-seven proteins were commonly found in both conditions, while 52 and 25 proteins were uniquely found in the LPS-stimulated and non-stimulated hemocytes, respectively. ATP-dependent energy-generating proteins such as actins and BLTX actin-related proteins were detected in both stimulated and non-stimulated *T*. *gigas* hemocytes, but more of such proteins were found in the former type. Proteins such as tachylectin-2, coagulogen, c-reactive proteins, histones, hemocyanin, and DNA polymerase, which play key roles in the organism’s innate immunity, were differentially expressed in the hemocytes following LPS challenge. In conclusion, the proteins identified in the hemolymph of *T*. *gigas* are vital for the organism’s molecular functions, biological processes, and activation of innate immunity.

## 1.Introduction

The taxon of invertebrates is the most diverse among animal taxa, and they have the most species. Invertebrates lack acquired immunity because they do not possess immune memory and antigen-antibody activity-based immune systems. Therefore, they heavily depend on the innate immune system to defend against invading pathogens [1]. The innate immune system of the invertebrates is domiciled in their circulatory system, usually the hemolymph [2]. Horseshoe crabs are one of the most studied invertebrates to understand the mechanisms and activities of innate immunity and other biological processes. The hemolymph of the horseshoe crabs contains hemocytes, which harbor many active biomolecules such as lectins, antimicrobial peptides, proteinase inhibitors and clotting factors [3]. The biomolecules are critical to the innate immune system of horseshoe crabs [3,4]. Decades ago, Marchalonins and Edelman [5] isolated and characterized limonin from the hemolymph of *Limulus polyphemus* (a species of horseshoe crab), which was proven to have the ability to recognize foreign cells and induce phagocytosis.

Moreover, tachyplesins have been found in the hemolymph of the *L*. *polyphemus*, *Tachypleus gigas*, *Tachypleus tridentatus and Carcinoscorpius rotundicauda* (South East Asian horseshoe crabs) [6–9]. Research revealed that these tachyplesins inhibit the growth of pathogenic microorganisms such as *Candida albicans*, and Gram-negative and Gram-positive bacteria [6–8].

In line with these discoveries and others, it is of great interest to profile the whole-proteome content of the horseshoe crab hemocytes to identify its proteins and investigate their biological activities. The hemocytes of the horseshoe crab are extremely sensitive to lipopolysaccharides (LPS), the cell wall component of Gram-negative bacteria [10]. Therefore, the present study profiled the proteins in the hemocytes of the Malaysian *T*. *gigas* under both LPS-induced and non-induced conditions.

## 2.Materials and methods

### 2.1 Experimental animals, hemocyte culture and LPS challenge

Adult horseshoe crabs (3) were collected from the Kuala Kemaman coastal region in Terengganu, Malaysia. The horseshoe crabs were captured at night and moved to the hatchery the next day, where they were acclimatized in three days. With the aid of pyrogen-free apparatus, the hemolymph (2 ml) was aseptically collected for each horseshoe crab. The collection was done using patented techniques (MY-155541-A) in a biological safety cabinet (ESCO, USA. A modified version of Osaki et al. [11] protocol recently reported by Samiento et al. [9] was adopted to carry out the LPS challenge on the hemolymph of the horseshoe crab.

### 2.2 Protein extraction from T. gigas hemocytes

The protocol reported by Niu et al. [12] was used for protein extraction and precipitation with some modifications. The supernatants were precipitated for 6 hours by adding 5 ml of ice-cold trichloroacetic acid (TCA)/acetone buffer (10% TCA (Merck) and 10 mM dithiothreitol (DTT)(Sigma-Aldrich) in acetone (Fisher Scientific)) to 1 ml of the sample. Then, the sample was centrifuged at 15,000 g for 10 min at 4°C, and the supernatant was discarded. The precipitated pellet was washed thrice with ice-cold 10 mM DTT (Sigma-Aldrich)-acetone (Fisher Scientific) solution and air-dried in a fume hood for 20–30 minutes. The protein pellet was resuspended in 300 μl of solubilization buffer [8 M urea (Sigma-Aldrich), 10 mM DTT (Sigma-Aldrich), 0.1 M triethylammonium bicarbonate (TEAB)(Sigma-Aldrich) at pH 8.5] by vortexing for 20 minutes. The sample was then centrifuged at 15,000 g for 15 min at 20°C twice, and the pellet was discarded.

### 2.3 Protein in-solution digestion

The non-stimulated (TgNS) and LPS stimulated (TgLPS) protein samples were separately digested by trypsinization using the protocol of Ru et al. [13]. Samples were reduced and alkylated before digestion. Briefly, protein samples were solubilized in 500μL of denaturing buffer [6M guanidine-HCl (Sigma-Aldrich)/25mM ammonium bicarbonate (Fisher Scientific), pH 8.5] and incubated with 250 μL of 1 mg/ml DTT (Sigma-Aldrich)/25 mM ammonium bicarbonate (Fisher Scientific) for 30 min at 55°C. Then, 500 μL of 1mg/mL iodoacetamide (Sigma-Aldrich)/25 mM ammonium bicarbonate (Fisher Scientific) was added, and the mixture was covered with aluminium foil and incubated for 15 min at 55°C. The reduced and alkylated protein sample went through buffer exchange with 25 mM ammonium bicarbonate (Fisher Scientific) using spin-columns with a molecular cut-off of 3 kDa (Merck Amicon Ultra 4) for 3 times. Then, 250 μL of digestion buffer (25 mM ammonium bicarbonate (Fisher Scientific)) and trypsin (Sigma-Aldrich) (ratio 1:50) were added to the sample and incubated at 37°C for 18 h. The samples were kept at -20°C or immediately analyzed.

### 2.4 Mass spectrometry

Liquid chromatography-tandem mass spectrometry (LC-MS/MS) analysis was performed using Linear Trap Quadrupole (LTQ) (Thermo Scientific, San Jose, CA, USA) coupled with Easy-nLC II system (Thermo Scientific, San Jose, CA, USA). Chromatographic separation of tryptic-digested peptides was performed using Easy-Column C18-A2 (100 × 0.75 mm i.d., 3 μm; Thermo Scientific, San Jose, CA, USA) coupled with pre-column (Easy-Column, 20 × 0.1 mm i.d., 5 μm; Thermo Scientific, San Jose, CA, USA) at the flow rate of 0.3 μl/min and sample injection volume of 10 μl. Running buffers used were: (A) deionized distilled water with 0.1% formic acid (Fluka Analytical) and (B) acetonitrile (Fisher Scientific) with 0.1% formic acid (Fluka Analytical). Gradient elution was carried out from 5 to 100% buffer B over 85 minutes and held for another 15 minutes. Data acquisition was carried out using Xcalibur ver. 2.1 (Thermo Scientific, San Jose, CA, USA) with a mass tolerance threshold of 5 ppm. The MS/MS analysis was carried out with collision-induced dissociation (CID) at 2 Da isolation width, activation *q* of 0.25, normalized collision energy of 35, 50 ms activation time and charge state of 2.

### 2.5 Analysis of mass spectrometric data

MS/MS data analysis was performed using PEAKS Studio v 7.5 (Bioinformatics Solutions Inc.). Precursor mass error tolerance 20 ppm using monoisotopic mass and fragment mass error tolerance 0.5 Da were set as the sequencing parameters with carbamidomethylation and oxidation (M) were set as fixed and variable modifications, respectively. The protein database from UniProt with taxonomy restriction of Arthropoda was used. The peptide-spectrum matches (PSM) results were filtered using high confidence peptide identification at 1% false rate discovery in all the workflow processes. The protein filter was set to -10lgP = 0 and 1 or more unique peptide hits.

### 2.6 Gene ontology analysis of horseshoe crab haemocyte proteins

Gene ontology (GO) analysis was performed on TgNS and TgLPS proteins after identification by PEAKS software. The GO annotation and KEGG [14] pathway analysis was performed on Blast2Go [15], and the graphical representation was performed on WEGO [16].

## 3.Results

### 3.1 Proteome of hemocytes of Tachypleus gigas

**Fig 1:**
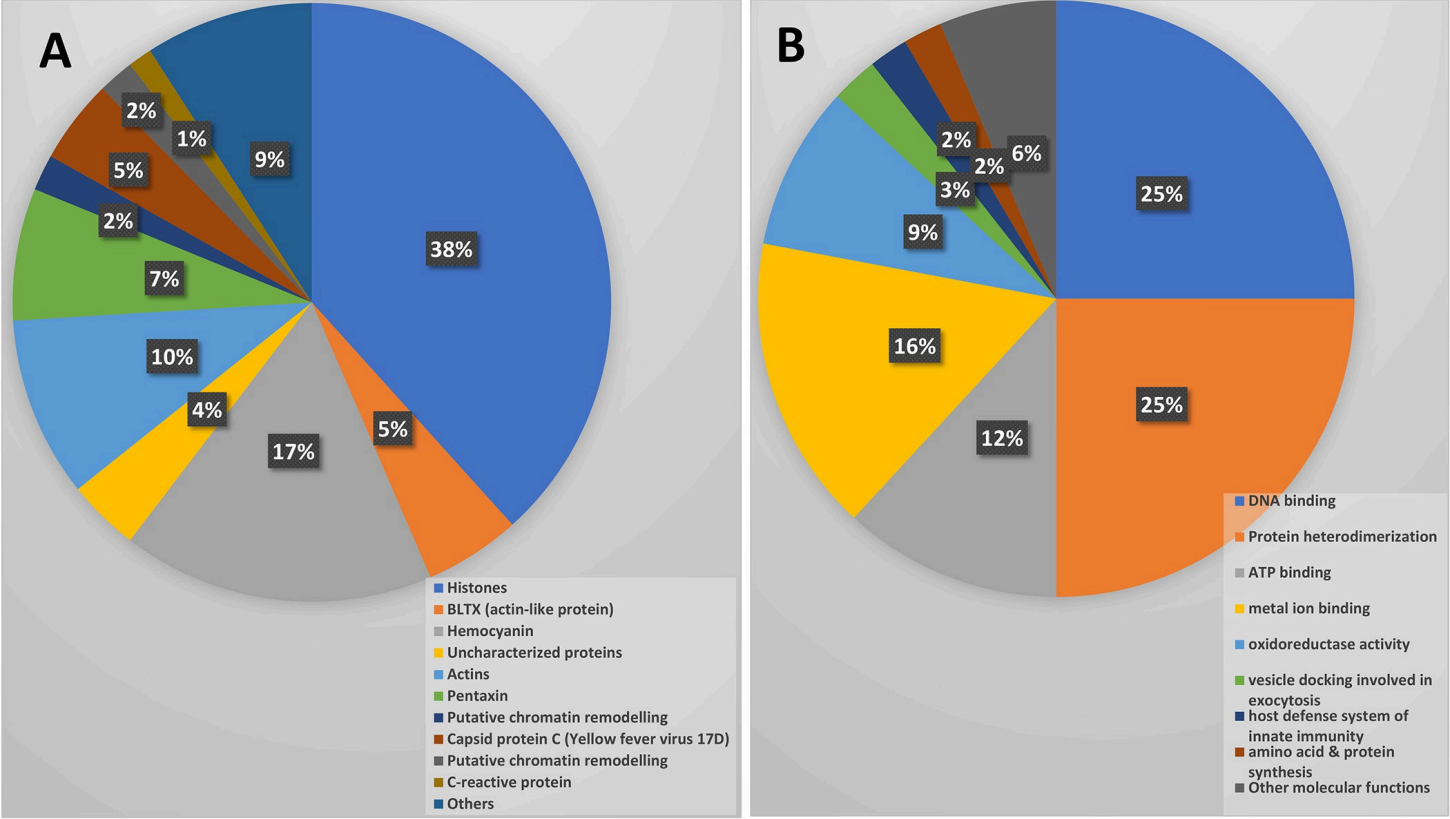
Combined classification of hemocytes proteins from LPS-stimulated and non-stimulated *T*. *gigas* samples. (A) Different types of proteins of the hemocytes of *T*. *gigas*. (B) The distribution of proteins with molecular and biological functions identified in the hemocytes of *T*. *gigas*.

The proteins of TgNS and TgLPS samples were extracted, and the proteins profiled using LC-MS/MS. To our knowledge, this is the first report on the whole proteome profiling of Malaysian *T*. *gigas* hemocytes. The LC-MS/MS protein profiling detected a total of 154 proteins in both TgNS and TgLPS hemocytes (Fig 1, S1 Table). Histones were found to be the most abundant protein with fifty-nine identities detected, representing 38% of the total protein component (S1 Table). Most of the histones identified were similar to those found in other invertebrates, especially other horseshoe crabs such as *Carcinoscorpius rotundicauda*, *Limulus Polyphemus*, *Tropilaelaps mercedesae* and *Nephila inaurata* as revealed by the UniProt database. The abundant proteins were hemocyanin (26; 17%) and actins (15; 10%). Eight actin-related blarina toxin (BLTX) proteins and 7 capsid proteins were also detected in the hemocytes (Fig 1A). We also identified 11 petaxins in the hemocytes of *T*. *gigas* that are identical to known petaxins of other invertebrates, and 6 uncharacterized proteins initially reported for *Tropilaelaps mercedesae*, *Amblyomma maculatum* and *Tetranychus urticae* (S1 Table). Other identified proteins include aspartate carbamoyltransferase catalytic subunit, gamma-glutamyl phosphate reductase, coagulogen, ABC transporter G family member 34, elongation factor G and GTP-binding protein Obg, among others (Fig 1A).

**Fig 2:**
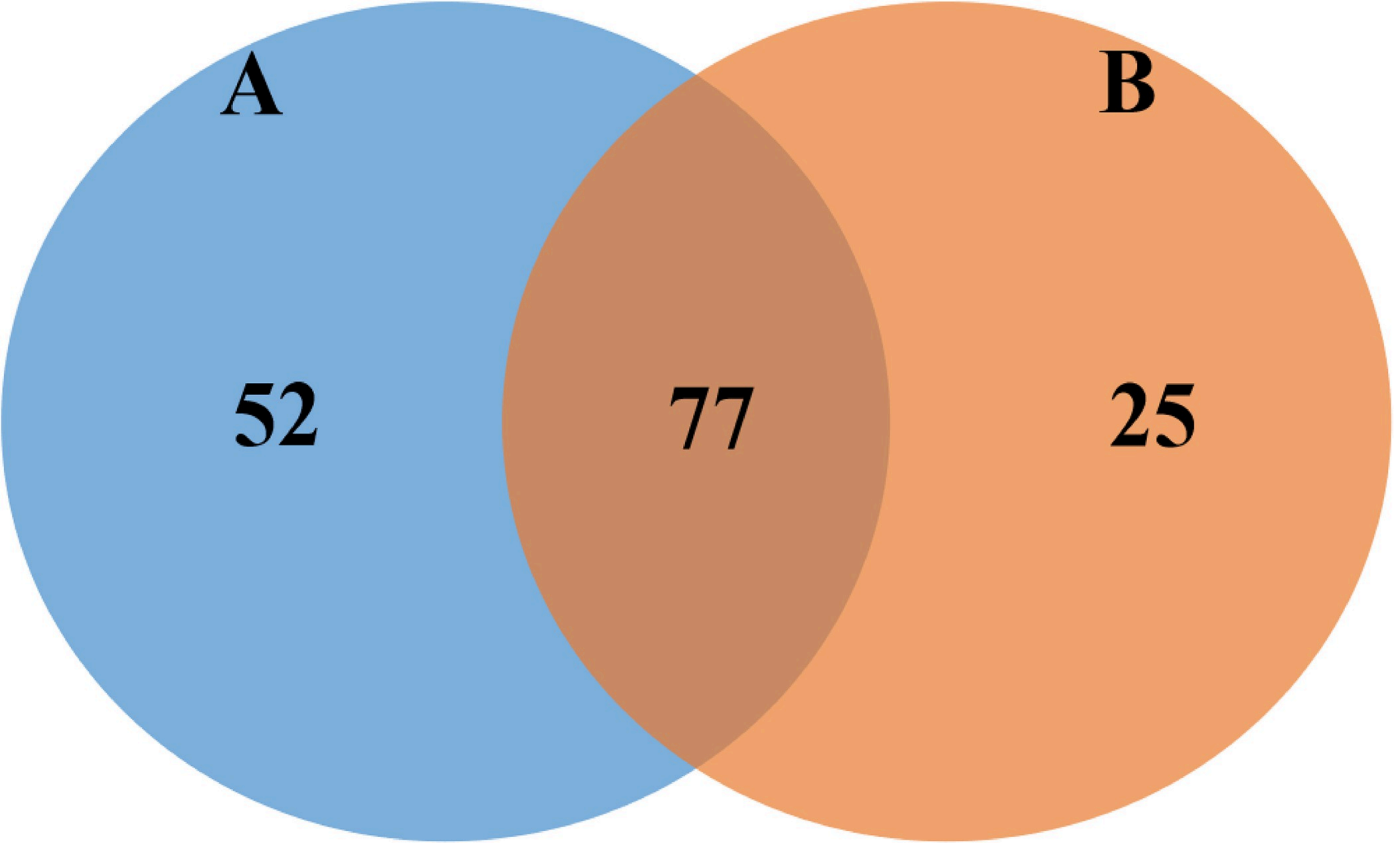
Comparison between the number of identified proteins in LPS-stimulated (A) and non-stimulated (B) hemocytes of *T*. *gigas*. A total number of 154 proteins were identified in both samples, 77 were commonly found in both samples. 52 proteins were uniquely found in the TgLPS, and 25 proteins were found in TgNS. TgNS, non-stimulated *T*. *gigas* hemocytes; TgLPS, lipopolysacharride-stimulated *T*. *gigas* hemocytes.

The general distribution of the molecular and biological functions of the identified proteins is displayed in Fig 1B. Most of the proteins are involved in DNA binding (25%), protein heterodimerization (25%), metal ion binding (16%) and ATP binding (12%). Oxidoreductase activity (9%), exocytosis vesicle docking (3%), and host innate immunity protein synthesis (2%) are part of other functions of the identified proteins. The total number of proteins identified in the hemocytes following LPS-stimulation was found to be increased. By comparison, seventy-seven proteins were commonly found in both TgLPS and TgNS samples. On the other hand, 52 and 25 proteins were uniquely found in the TgLPS and TgNS hemocytes, respectively (Fig 2).

### 3.2 Comparative gene ontology analyses of T. gigas hemocyte proteins following LPS challenge

**Fig 3:**
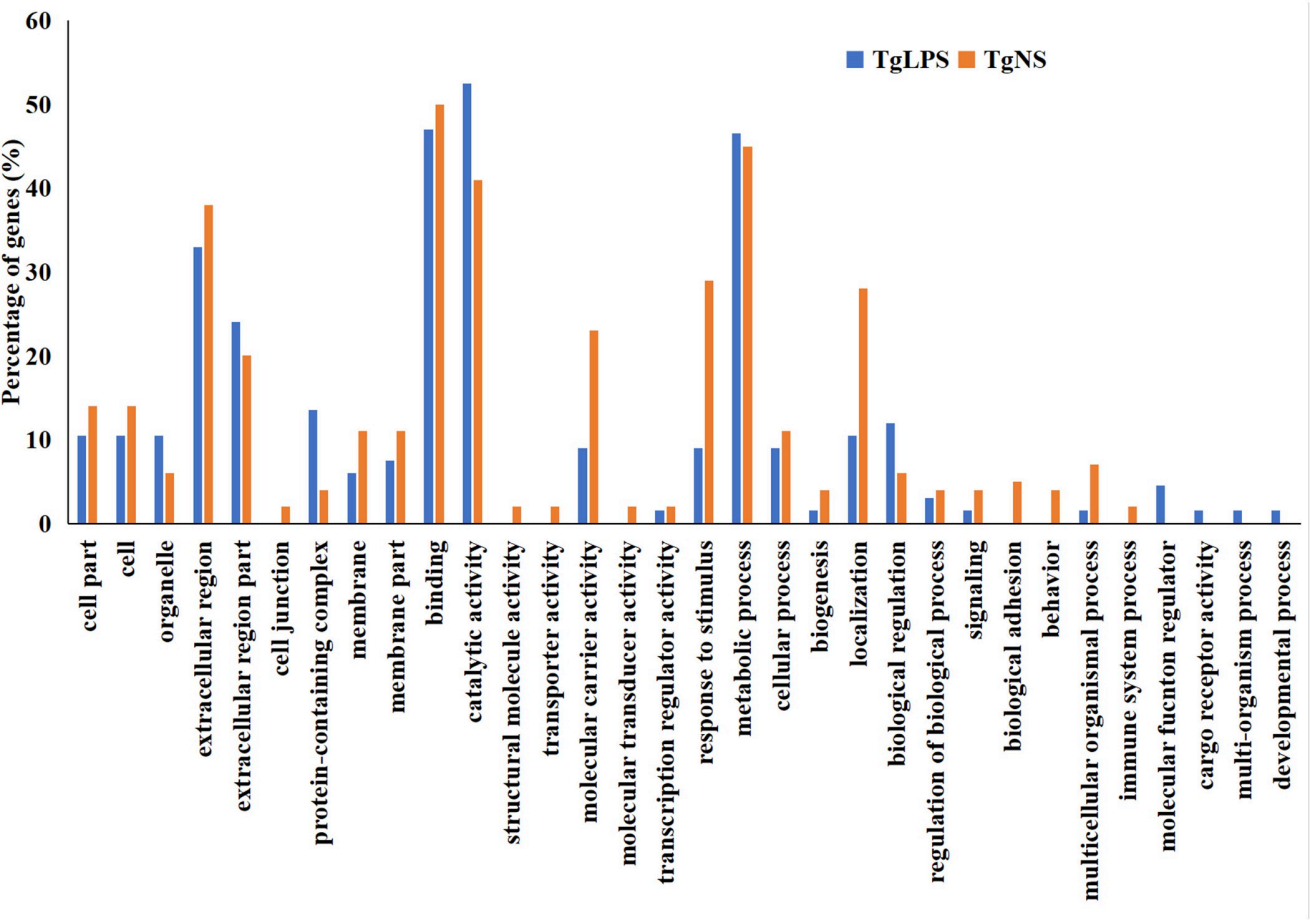
The gene ontology of the proteins identified in TgNS and TgLPS. The analysis was performed using PEAKS software. These proteins are either cellular components, have specific molecular functions or are involved in the biological processes. TgNS, non-stimulated *T*. *gigas* hemocytes; TgLPS, lipopolysacharride-stimulated *T*. *gigas* hemocytes.

Gene ontology analysis of the profiled proteins in TgNS and TgLPS samples were carried out, and the results are presented in Fig 3. The identified proteins of the hemocytes of *T*. *gigas* before and after LPS challenge are associated with various molecular and biological functions of the organism. In terms of cellular components, the percentage of functional genes involved in cells, extracellular regions, cell junction and cell membrane component formation are higher in TgNS than in TgLPS. On the other hand, the percentage of functional genes involved in organelle and protein-containing complex component composition is elevated following LPS stimulation.

Regarding the molecular function, the genes involved in binding activities, structural molecule activity, transporter activity, molecular carrier and transducer activities, and transcription regulator activity are more abundant in TgNS than in TgLPS. Meanwhile, the expression of catalytic activity-related genes is higher in TgLPS than in TgNS. On biological processes, the expression of genes that are involved in stimulus responses, biogenesis, localization, signaling, biological adhesion, behavior, multicellular organismal process, and immune system processes is higher in TgNS than in TgLPS. On the other hand, genes involved in biological regulation are more expressed in TgLPS than TgNS. The expression level of genes involved in other biological processes are about the same in both experimental conditions.

KEGG pathway analysis revealed that one protein from each TgNS and TgLPS are a component of several biological pathways (S1–S7 Figs). The phosphatase protein uniquely identified in TgNS is involved in purine metabolism pathway (KEGG pathway ID: map00230) and thiamine metabolism pathway (KEGG pathway ID: map00730). The hydroxymethyltransferase protein, which was uniquely identified in TgLPS, is involved in one-carbon pool by folate pathway (KEGG pathway ID: map00670), glyoxylate and dicarboxylate metabolism pathway (KEGG pathway ID: map00630), glycine, serine and threonine metabolism pathway (KEGG pathway ID: map00260), methane metabolism pathway (KEGG pathway ID: map00680), and cyanoamino acid metabolism pathway (KEGG pathway ID: map00460).

### 3.3 Changes in expression/abundance of proteins of T. gigas hemocytes following LPS stimulation

**Fig 4:**
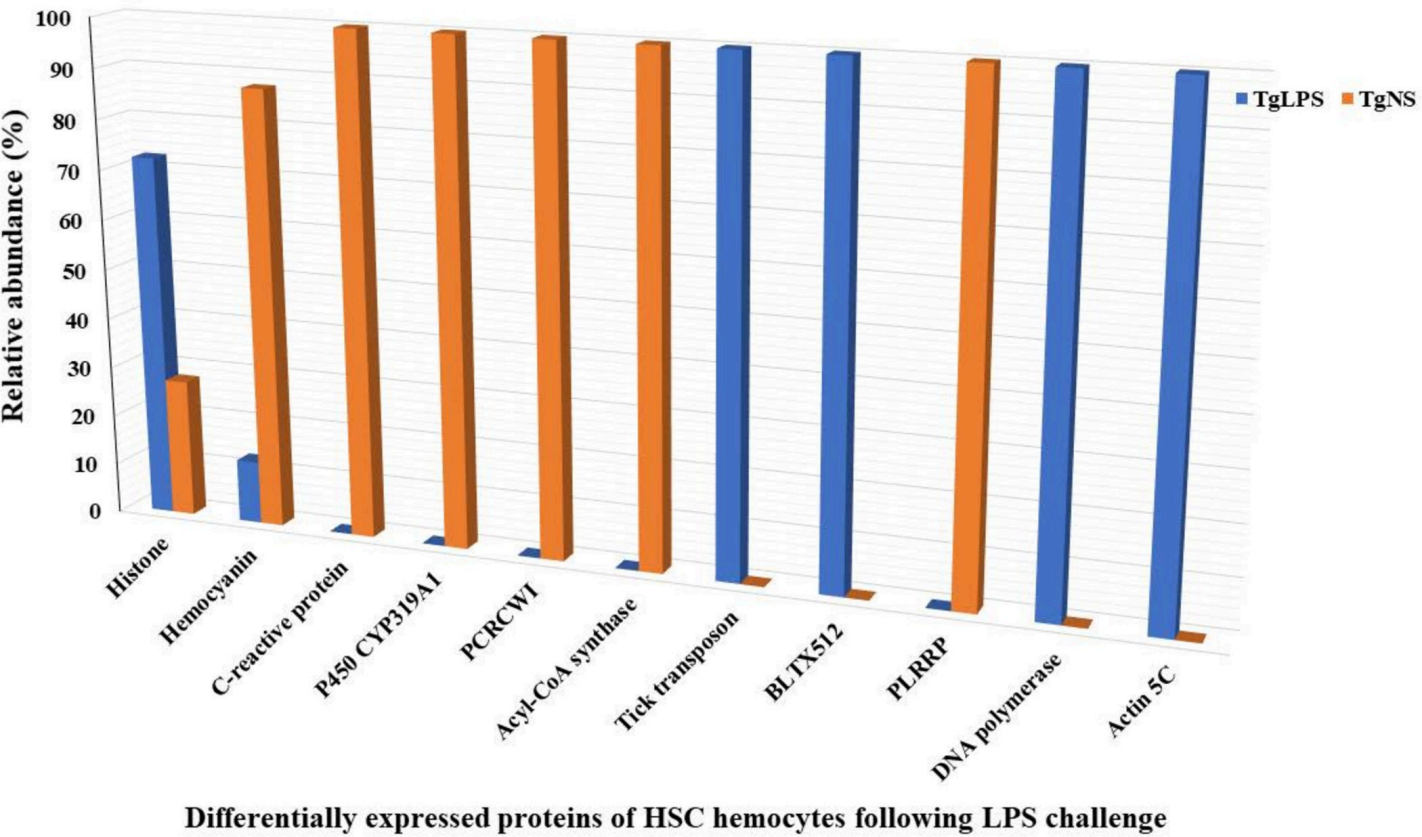
*T*. *gigas* proteins that are differentially expressed after LPS challenge. As displayed in the figure, the expression/abundance of proteins of the hemocytes of *T*. *gigas* were altered which signify the effect of the LPS challenge (mimics of bacterial infection). PCRWI- putative chromatin remodeling complex wstf-iswi, PLRRP- putative leucine-rich repeat protein. TgNS, non-stimulated *T*. *gigas* hemocytes and TgLPS, lipopolysacharride-stimulated *T*. *gigas* hemocytes.

Following LPS challenge, the expression and abundance of some proteins were altered. The expressions and abundance of histones, DNA polymerase, Actin5C, BLTX512, and tick transposon were increased after LPS was introduced to *T*. *gigas* hemocytes. On the other hand, the expression and abundance of hemocyanin, CRP, acetyl Co-A synthase, P450 CYP319A1, putative chromatin remodeling complex wstf-iswi, and putative leucine-rich repeat protein decreased after LPS was introduced to *T*. *gigas* hemocytes (Fig 4).

## 4.Discussion

### 4.1 Proteins involved in the biological processes of T. gigas

Some of the proteins found in TgLPS and TgNS hemocytes are important in the biological activities of the animal. The detection of actins in the hemocytes is of no surprise as these proteins are housekeeping proteins that are naturally domiciled in the hemocytes of horseshoe crabs [17]. However, the results revealed the expression of more actin-related proteins in the LPS-stimulated hemocytes, which indicates that the horseshoe crab is more energized to execute its defense system to fight against external pathogens, as actins are involved in ATP production [18]. Proteins involved in defending the host against external pathogens, such as tachylectin and coagulogen, were identified in both LPS-stimulated and non-stimulated conditions [19]. In corroboration with this study, Sarmiento et al. (2021) also detected tachylectins and coagulogen in the hemocytes of the horseshoe crab at transcriptomic mRNA levels, and the expressions of the lectin genes were altered when the homocytes was exposed to LPS [20]. This indicates that these proteins used for host defense are readily synthesized and available in the hemocytes before exposure to pathogens.

The study revealed that hemocytes are rich in energy-generating molecules as many proteins that function as ATP binding factors were detected, including actins, BLTX-actin-related proteins, and some proteins that were yet to be characterized (Fig 1) [18,21]. Petaxins, putative chromatin remodelling complex wst-iswi, c-reactive proteins (CRP), cationic trypsin, and hemocyanin form part of the housekeeping proteins of the hemocytes and are involved in metal ion binding [22,23]. Hemocyanin is present in the hemocytes of mollusks and arthropods, including horseshoe crabs [24]. In addition, hemocyanin plays key roles in oxidoreductase activity and the host defense system innate immunity of horseshoe crabs (Fig 1B) [25]. Similarly, hemocyanin gene was also detected in the transcriptomic sample of the *T*. *gigas* hemocytes [20].

Some detected proteins are involved in DNA replication and transcription, RNA translation and protein biosynthesis (Fig 1B). Histones are involved in DNA binding and protein heterodimerization. DNA polymerase was also identified, which catalyzes the addition of new nucleotides for elongation of DNA strands during DNA replication [26]. Supportively, the DNA polymerase gene was also detected at transcriptomic level when LPS was exposed to the hemocytes of *T*. *gigas* [20]. Aspartate carbamoyltransferase catalyzes amino acid binding during translation of RNA, and it is involved in the metabolic process of cellular amino acid and pyrimidine biosynthesis [27]. Gamma-glutamyl phosphate reductase plays a significant role in L-proline biosynthesis by catalyzing the L-glutamate 5-phosphate reduction into phosphate and L-glutamate 5-semialdehyde [28]. Elongation factor G has a necessary action during protein translation elongation and protein biosynthesis [29]. GTPase Obg was also found in the hemolymph, and it is also involved in ribosome biogenesis [29].

Interestingly, KEGG pathway analysis revealed two of the *T*. *gigas* proteins (phosphatase and hydroxymethyltransferase) are involved in several biological pathways which are originally known to be found in higher vertebrate animals. The fact that the proteins are also found in invertebrate hemocytes could give us insight into the unknown relationship which may exist between invertebrates and vertebrates in terms of biological processes and molecular activities and functions. This is an important aspect worth exploring.

Alpha-2-macroglobulin, which has endopeptidase inhibitor activity, was also detected in the hemocytes [30]. Tachylectin-2, CRPs, hemocyanins, ABC transporter G family member 34 and coagulogen play distinctive roles in the innate immunity of the host defense system of horseshoe crabs. Details on their functions are discussed in the following subsection.

### 4.2 Proteins that are involved in the host defense system of T. gigas

Several proteins were detected in the hemocytes of the horseshoe crabs, which could be responsible for the organism’s innate immunity. Tachylectin-2 is a type of lectin that has been previously identified in the hemocytes of other horseshoe crabs, such as the Japanese *T*. *tridentatus*. It has a hemagglutinating effect and antimicrobial activity against many microorganisms such as *E*. *coli*, *S*. *Minnesota*, *P*. *auruginosa*, *K*. *pneumoniae*, *S*. *flexneri*, *S*. *aureus*, *S*. *sanguis*, *S*. *pyogenes*, *B*. *subtilis* and *S*. *mutans* [10].

Coagulogen is an essential hemolymph protein, and it is an integral part of horseshoe crabs’ host defence system. Once the organism senses molecules of pathogens, it triggers the formation of a gel produced by the cleavage of coagulogen to give coagulin. The formed coagulin gel then binds with proxins to form a polymer matrix that encapsulates the pathogen to immobilize it and inhibit its growth and proliferation [11,31]. C-Reactive proteins (CRP) were also identified, and they have been reported to play a pivotal role in the innate immunity of horseshoe crab against pathogenic microorganisms [32]. It acts by recognizing and binding with the LPS of the pathogenic organisms. The interaction recruits phagocytic cells to clear the pathogens in the system. *In vivo* study conducted by Ng et al. [33] revealed the CRP binds with *P*. *aeruginosa* to inhibit and kill the organisms.

Meanwhile, several researchers found that histones could inhibit and kill gram-negative bacteria in horseshoe crab hemocytes as their expression was found to be upregulated when infected [34,35]. Similarly, an increase in the concentration of alpha-2-macroglobulin was reported in the hemocytes in response to gram-negative bacteria infection [36].

Aspartate carbamoyltransferase catalytic subunit synthesizes pyrimidines, known antimicrobial agents [37]. Meanwhile, gamma-glutamyl phosphate reductase plays a vital role in the biosynthesis of proline. Some proline-rich peptides possess antimicrobial properties [38–40]. On the other hand, hemocyanin is converted to phenoloxidase upon the action of coagulation factor B and is involved in the detection and killing of microorganisms and the production of melanin involved in wound healing and encapsulation of microorganisms [41–43]. Hence, hemocyanins are found to possess antibacterial effects [44]. In conclusion, the proteins identified in the hemolymph of *T*. *gigas* are vital for the molecular functions, biological processes, and activation of innate immunity of Malaysian *T*. *gigas*.

### 4.3 Implication of bacterial infection mimics LPS challenge on the abundance/expression of proteins in the HSC hemocytes

The LPS was introduced to the isolated *T*. *gigas* hemocytes to mimic a scenario of bacterial infection and stimulate the innate immune response of the *T*. *gigas* through its hemocytes. Expectedly, antimicrobial proteins, CRP and hemocyanin were found to be degraded or reduced in expression following the LPS challenge (Fig 4). This reduction can be linked to their antimicrobial activity as they are expected to degrade and chemically metamorphose while they exhibit their inhibitory or killing effects on invading pathogens as earlier mentioned [32,44].

Also, histones were found to have higher expression and abundance in the LPS stimulated hemocytes (Fig 4) as compared to non-stimulated hemocytes. This observation clearly revealed that the histones were responding to the sensed invading agents. The results conform with previous reports which stated that an increase in histone expression in invertebrates’ hemocytes correspond to their effects of inhibiting and killing of bacterial pathogens [34,35].

Acetyl Co-A synthase was detected in TgNS sample but not in TgLPS sample. Acetyl Co-A synthase catalyzes the synthesis of acetyl Co-A,an essential precursor for ATP energy generation [45]. Hence, the unavailability of acetyl Co-A synthase in TgLPS implies that it could have been used up following the LPS challenge for the generation of energy needed to fight off the invading agent (LPS). Similarly, the putative chromatin remodelling complex wstf-iswi was found in TgNS sample but not TgLPS sample. This protein functions as an ATP binding molecule involved in nucleosome-dependent ATPase activity [46,47]. This adds to the likelihood that energy was expended to defend against the invading agent (LPS).

The BLTX512 and actin5C were exclusively identified in TgLPS hemocytes but not TgNS samples. These two proteins are members of actin-related proteins; even though their specific roles in *T*. *gigas* hemocytes are yet to be known, actins are generally involved in multiple cellular functions such as ATP binding, cell motility, chromosome movement and cytoskeleton structure [48,49].

The detection of DNA polymerase in TgLPS sample, which was not found in TgNS sample, could imply the synthesis of more hemocytes in the hemolymph of *T*. *gigas*. The additional hemocytes could be needed to challenge the perceived invading agent since DNA polymerase is the primary enzyme required for DNA replication, an essential initial process for cell growth and multiplication [50]. The absence of the chromatin remodeling complex in TgLPS even when DNA polymerase was founud could be because of the formation of chromatin fibers immediately after DNA replication, and hence the formation of heterochromatin which is transcriptionally inactive [51]. This could limit excessive expression of proinflammatory genes [51].

Tick transposon was exclusively found in TgLPS sample. On the other hand, putative P450CYP319A1 and putative leucine-rich repeat protein were uniquely found in TgNS sample. The roles and functions of these proteins in response to the bacterial infection are worth investigating.

## 5.Conclusion

In conclusion, the proteins identified in the hemolymph of *T*. *gigas* are vital for the organism’s molecular functions, biological processes, and activation of innate immunity. In addition, the bacterial-mimic LPS challenge to *T*. *gigas* hemocytes provides insight into the organism’s innate immune system, which could be useful in future human applications. Further quantitation studies need to be conducted to unravel the immune system’s mechanism of *T*. *gigas*.

## Supporting information

S1 Fig Purine metabolism pathway. (PDF)

S2 Fig Thiamine metabolism pathway. (PDF)

S3 Fig One carbon pool by folate pathway. (PDF)

S4 Fig Glyoxylate and dicarboxylate metabolism pathway. (PDF)

S5 Fig Glycine, serine, and threonine metabolism pathway. (PDF)

S6 Fig Methane metabolism pathway. (PDF)

S7 Fig Cyanoamino acid metabolism pathway. (PDF)

S1 Table List of proteins identified in TgLPS and TgNS samples. (PDF)
