# Supplementary material for: Proteomic analysis of Malaysian Horseshoe crab (Tachypleus gigas) hemocytes gives insights into its innate immunity host defence system and other biological processes
Source: PLoS One. 2022 Aug 10;17(8):e0272799. doi: 10.1371/journal.pone.0272799 (PMC9365167; doi:10.1371/journal.pone.0272799)
Supplement: S4 Fig — (PDF) [file pone.0272799.s004.pdf]

Supplementary Figure 4: Glyoxylate and Dicarboxylate Metabolism Pathway

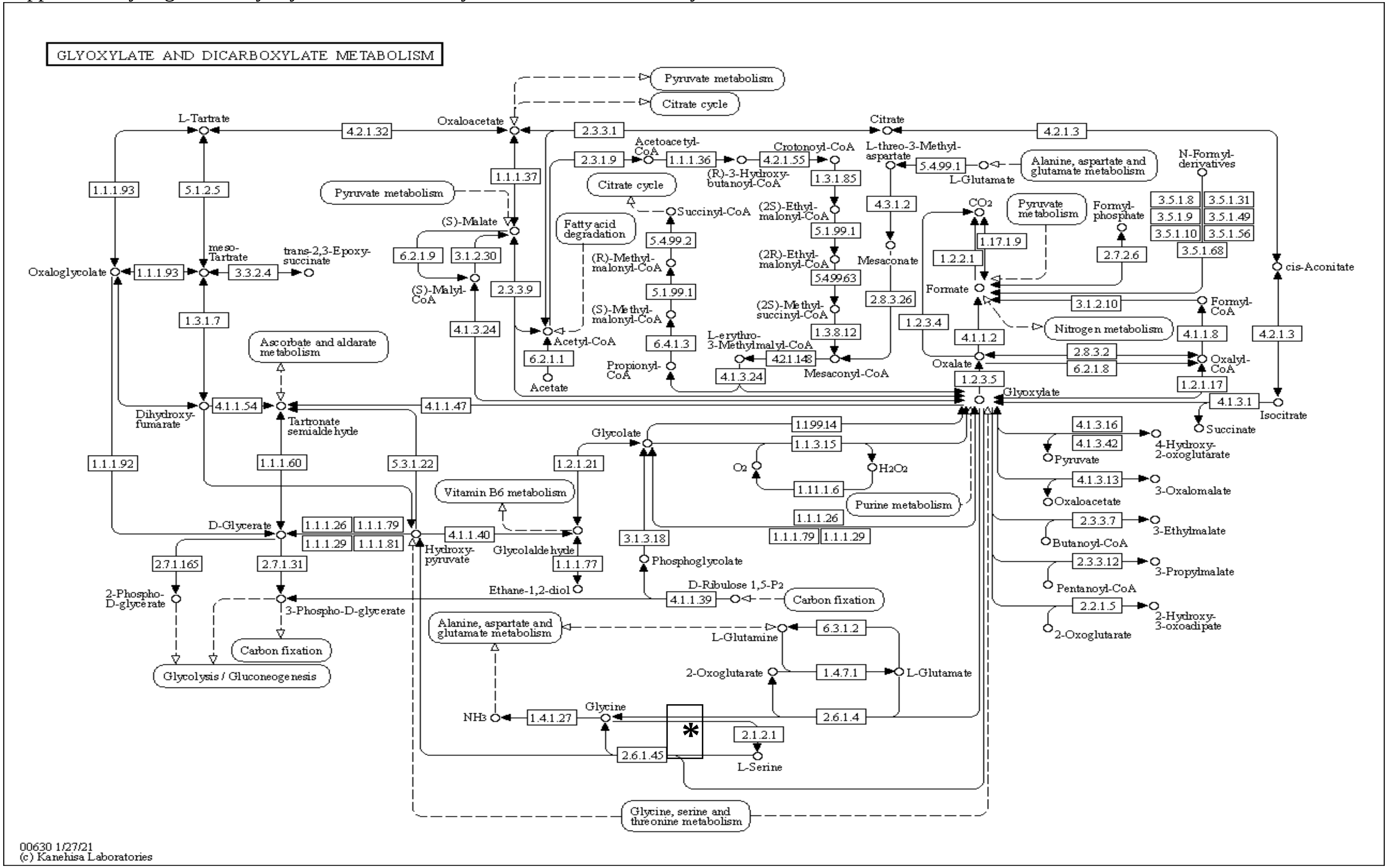

Source: https://www.genome.jp/kegg/kegg2.html. The position of hydroxymethyltransferase in the pathway is signified by '*'
