# Supplementary material for: Proteomic analysis of Malaysian Horseshoe crab (Tachypleus gigas) hemocytes gives insights into its innate immunity host defence system and other biological processes
Source: PLoS One. 2022 Aug 10;17(8):e0272799. doi: 10.1371/journal.pone.0272799 (PMC9365167; doi:10.1371/journal.pone.0272799)
Supplement: S1 Table — (PDF) [file pone.0272799.s008.pdf]

Supplementary Table 1. list of proteins identified in TgLPS and TgNS samples

| | | LPS stimulated sample [2018-10-26 09:33] | | | | | | Non-stimulated sample [2018-10-26 09:33] | | | | | | Protein name |
|---|---|---|---|---|---|---|---|---|---|---|---|---|---|---|
| Protein_ID | Cover Map* | Score | #Spec | #Pep | #Uniq | %Spec | %Cov | Score | #Spec | #Pep | #Uniq | %Spec | %Cov | Protein name |
| tr\|T1Z0D0\|T1Z0D0_9ARAC | + - | 33.69 | 1 | 1 | 1 | - | 8.33 | | | | | | | Histone H3. *Nephila inaurata (golden orb- web spider)* |
| tr\|A0A023G9L8\|A0A023G9L8_9ACAR | + - | 53.74 | 2 | 1 | 1 | - | 6.25 | | | | | | | Histone H2A. *Amblyomma triste* |
| tr\|A0A076KU51\|A0A076KU51_NEPPI | + + | 89.3 | 3 | 2 | 1 | - | 9.38 | 118.9 | 4 | 2 | 2 | - | 9.38 | BLTX520. *Nephila pilipes (Giant wood spider) (Nephila maculata)* |
| tr\|A2AX56\|A2AX56_LIMPO | - + | | | | | | | 118.09 | 6 | 4 | 1 | - | 7.95 | Hemocyanin subunit II ( *Limulus polyphemus (Atlantic horseshoe crab)* |
| tr\|A0A1V9XXZ1\|A0A1V9XXZ1_9ACAR | - + | | | | | | | 70.48 | 3 | 2 | 2 | - | 8.24 | Histone H4 (Tropilaelaps mercedesae) |
| tr\|A2AX59\|A2AX59_LIMPO | + + | 67.38 | 2 | 1 | 1 | - | 2.35 | 142.23 | 8 | 5 | 3 | - | 12.07 | Hemocyanin subunit VI OS=Limulus polyphemus GN=hcVI PE=2 SV=1 |

| | | | | | | | | | | | | | |
|---|---|---|---|---|---|---|---|---|---|---|---|---|---|
| tr\|A0A125RQ50\|A0A125RQ50_9ARAC | + - | 33.69 | 1 | 1 | 1 | - | 7.76 | | | | | | Histone H3. *Caerostris sexcuspidata* |
| tr\|A1X1V1\|A1X1V1_CARRO | - + | | | | | | | 185.96 | 9 | 7 | 1 | - | 13.46 | Hemocyanin subunit I OS=Carcinoscorpius rotundicauda PE=2 SV=1 |
| sp\|Q27084\|TAL2_TACTR | + + | 42.65 | 3 | 1 | 1 | - | 3.14 | 44.61 | 2 | 1 | 1 | - | 3.14 | Tachylectin-2 |
| tr\|G3MKZ5\|G3MKZ5_9ACAR | + - | 53.74 | 2 | 1 | 1 | - | 6.25 | | | | | | | Histone H2A OS=Amblyomma maculatum PE=2 SV=1 |
| tr\|A3QQG8\|A3QQG8_9CHEL | + - | 33.69 | 1 | 1 | 1 | - | 8.33 | | | | | | | dofleini GN=H3 PE=3 SV=1 |
| tr\|A0A224Z0J6\|A0A224Z0J6_9ACAR | + + | 53.74 | 2 | 1 | 1 | - | 7.26 | 79.56 | 3 | 2 | 2 | - | 33.87 | Histone H2A. *Rhipicephalus zambeziensis* |
| tr\|A0A0D3QJ45\|A0A0D3QJ45_PANCT | + + | 89.3 | 3 | 2 | 1 | - | 7.18 | 118.9 | 4 | 2 | 2 | - | 7.18 | Actin. *Panonychus citri (Citrus red mite) (Tetranychus citri)* |
| tr\|I2A6L7\|I2A6L7_9ARAC | + - | 33.69 | 1 | 1 | 1 | - | 8.26 | | | | | | | Histone H3 (Fragment) OS=Zalmoxis sp. DNA102357-2 GN=H3 PE=3 SV=1 |
| tr\|G8I0T8\|G8I0T8_9ARAC | + - | 33.69 | 1 | 1 | 1 | - | 8.26 | | | | | | | Histone H3 (Fragment) OS=Parachtes romandiolae GN=H3 PE=3 SV=1 |

| | | | | | | | | | | | | | | | |
|---|---|---|---|---|---|---|---|---|---|---|---|---|---|---|---|
| tr\|H8Y6F6\|H8Y6F6_9ARAC | + - | 33.69 | 1 | 1 | 1 | - | 8.26 | | | | | | | Histone H3 (Fragment) OS=Phyxelida bifoveata GN=H3 PE=3 SV=1 |
| tr\|A0A293LRQ2\|A0A293LRQ2_ORNER | + + | 53.74 | 2 | 1 | 1 | - | 7.26 | 79.56 | 3 | 2 | 2 | - | 33.87 | Histone H2A. *Ornithodoros erraticus (European soft tick) (Alectorobius erraticus)* |
| tr\|A0A076KU31\|A0A076KU31_NEPPI | + + | 89.3 | 3 | 2 | 1 | - | 7.94 | 118.9 | 4 | 2 | 2 | - | 7.94 | BLTX498. *Nephila pilipes (Giant wood spider) (Nephila maculata)* |
| tr\|A0A132AEN9\|A0A132AEN9_SARSC | + + | 53.74 | 2 | 1 | 1 | - | 10.23 | 79.56 | 3 | 2 | 2 | - | 47.73 | Histone H2A. *Sarcoptes scabiei (Itch mite) (Acarus scabiei)* |
| tr\|O01717\|O01717_9CHEL | + + | 85.39 | 7 | 4 | 4 | - | 2.92 | 117.58 | 24 | 8 | 8 | - | 4.05 | Alpha-2-macroglobulin ( *Limulus sp.)* |
| tr\|A0A076L0D3\|A0A076L0D3_NEPPI | + + | 89.3 | 3 | 2 | 1 | - | 8.71 | 118.9 | 4 | 2 | 2 | - | 8.71 | BLTX538. *Nephila pilipes (Giant wood spider) (Nephila maculata)* |
| tr\|G8I0T1\|G8I0T1_9ARAC | + - | 33.69 | 1 | 1 | 1 | - | 9 | | | | | | | Histone H3. *Parachtes ignavus* |
| tr\|A0A1V9X8H0\|A0A1V9X8H0_9ACAR | - + | | | | | | | 29.42 | 1 | 1 | 1 | - | 5.5 | Uncharacterized protein. *Tropilaelaps mercedesae.* |
| tr\|A0A1W7RAE8\|A0A1W7RAE8_9SCOR | - + | | | | | | | 104.11 | 5 | 3 | 1 | - | 4.44 | Hemocyanin subunit 5b. |

| | | | | | | | | | | | | | | |
|---|---|---|---|---|---|---|---|---|---|---|---|---|---|---|
| | | | | | | | | | | | | | | *Hadrurus spadix* |
| tr\|S5G8M7\|S5G8M7_9ACAR | + + | 89.3 | 3 | 2 | 1 | - | 7.18 | 118.9 | 4 | 2 | 2 | - | 7.18 | Actin 1 OS = Neoseiulus cucumeris PE=2 SV=1 |
| tr\|A0A076KV49\|A0A076KV49_NEPPI | + + | 89.3 | 3 | 2 | 1 | - | 8.79 | 118.9 | 4 | 2 | 2 | - | 8.79 | BLTX569. *Nephila pilipes (Giant wood spider) (Nephila maculata)* |
| tr\|A0A224YZ06\|A0A224YZ06_9ACAR | + - | 53.74 | 2 | 1 | 1 | - | 7.03 | | | | | | | Histone H2A. *Rhipicephalus zambeziensis* |
| tr\|B7QBU0\|B7QBU0_IXOSC | - + | | | | | | | 24.47 | 1 | 1 | 1 | - | 3.02 | P450 CYP319A1, putative (Fragment) (Fragment). OS = *Ixodes scapularis (Black-legged tick) (Deer tick)* |
| tr\|Q9U8Z5\|Q9U8Z5_TACTR | + + | 93.39 | 8 | 3 | 3 | - | 13.86 | 98.6 | 15 | 2 | 1 | - | 13.86 | Pentaxin OS= *Tachypleus tridentatus* (Japanese horseshoe crab) |
| tr\|A0A0S1LJA2\|A0A0S1LJA2_9ARAC | + - | 33.69 | 1 | 1 | 1 | - | 8.26 | | | | | | | Histone H3. *Gamakia hirsute* |

| | | | | | | | | | | | | | | | |
|---|---|---|---|---|---|---|---|---|---|---|---|---|---|---|---|
| tr\|A0A023FHH5\|A0A023FHH5_9ACAR | + + | 53.74 | 2 | 1 | 1 | - | 7.26 | 79.56 | 3 | 2 | 2 | - | 33.87 | Histone H2A. *Amblyomma cajennense (Cayenne tick)* |
| tr\|D0VSU5\|D0VSU5_9ARAC | + - | 33.69 | 1 | 1 | 1 | - | 8.26 | | | | | | | Histone H3 (Fragment) OS=Selenops lindborgi GN=H3a PE=3 SV=1 |
| tr\|A0A1I9J789\|A0A1I9J789_9ARAC | + - | 33.69 | 1 | 1 | 1 | - | 8.26 | | | | | | | Histone H3. *Synotaxidae sp. LB- 2014a* |
| tr\|A0A023GJC4\|A0A023GJC4_9ACAR | + + | 89.3 | 3 | 2 | 1 | - | 7.18 | 118.9 | 4 | 2 | 2 | - | 7.18 | Putative actin. *Amblyomma triste* |
| tr\|A0A125RQ54\|A0A125RQ54_9ARAC | + - | 33.69 | 1 | 1 | 1 | - | 7.76 | | | | | | | Histone H3. *Guizygiella nadleri* |
| tr\|A0A1E1XAH7\|A0A1E1XAH7_9ACAR | - + | | | | | | | 31.59 | 1 | 1 | 1 | - | 1.8 | Putative chromatin remodeling complex wstf-iswi. *Amblyomma aureolatum* |
| tr\|A0A1E1X1E7\|A0A1E1X1E7_9ACAR | + - | 53.74 | 2 | 1 | 1 | - | 6.25 | | | | | | | Histone H2A. *Amblyomma aureolatum* |
| tr\|A0A023GER7\|A0A023GER7_9ACAR | + + | 53.74 | 2 | 1 | 1 | - | 6.52 | 74.19 | 3 | 2 | 2 | - | 30.43 | Histone H2A. *Amblyomma triste* |
| tr\|A2AX57\|A2AX57_LIMPO | - + | | | | | | | 179.25 | 9 | 6 | 4 | - | 10.05 | Hemocyanin subunit IIIa OS=Limulus polyphemus GN=hcIIIa PE=2 SV=1 |

| | | | | | | | | | | | | | | | |
|---|---|---|---|---|---|---|---|---|---|---|---|---|---|---|---|
| tr\|T1D1T4\|T1D1T4_CUPSA | + + | 89.3 | 3 | 2 | 1 | - | 7.18 | 118.9 | 4 | 2 | 2 | - | 7.18 | T1D1T4 Putative actin OS=Cupiennius salei PE=2 SV=1 |
| tr\|A0A034WXE4\|A0A034WXE4_RHIMP | + + | 53.74 | 2 | 1 | 1 | - | 7.26 | 79.56 | 3 | 2 | 2 | - | 33.87 | Histone H2A. *Rhipicephalus microplus (Cattle tick) (Boophilus microplus)* |
| tr\|A0A1E1X3F6\|A0A1E1X3F6_9ACAR | + + | 89.3 | 3 | 2 | 1 | - | 7.18 | 118.9 | 4 | 2 | 2 | - | 7.18 | Putative actin related protein 1. *Amblyomma aureolatum* |
| sp\|P06207\|CRP3_LIMPO | - + | | | | | | | 115.08 | 13 | 3 | 2 | - | 15.29 | C-reactive protein 3.3. OS = *Limulus polyphemus (Atlantic horseshoe crab)* |
| sp\|P06205\|CRP1_LIMPO | - + | | | | | | | 115.08 | 13 | 3 | 2 | - | 15.29 | C-reactive protein 1.1. OS = *Limulus polyphemus (Atlantic horseshoe crab)* |
| tr\|A0A023FGQ4\|A0A023FGQ4_9ACAR | + + | 53.74 | 2 | 1 | 1 | - | 7.26 | 79.56 | 3 | 2 | 2 | - | 33.87 | Histone H2A. *Amblyomma cajennense (Cayenne tick)* |
| tr\|A2AX58\|A2AX58_LIMPO | + + | 93.52 | 5 | 3 | 1 | - | 5.13 | 182.74 | 13 | 8 | 2 | - | 16.35 | Hemocyanin subunit IV OS=Limulus polyphemus GN=hcIV PE=2 SV=1 |
| tr\|A0A2I4K5B6\|A0A2I4K5B6_9ACAR | + + | 53.74 | 2 | 1 | 1 | - | 6.52 | 74.19 | 3 | 2 | 2 | - | 30.43 | Histone H2A. *Rhipicepha lus* |

| | | | | | | | | | | | | | | |
|---|---|---|---|---|---|---|---|---|---|---|---|---|---|---|
| | | | | | | | | | | | | | | *annulatus* |
| tr\|Q9U8Z9\|Q9U8Z9_TACTR | + + | 93.39 | 8 | 3 | 3 | - | 13.86 | 98.6 | 15 | 2 | 1 | - | 13.86 | Pentaxin. OS= *Tachypleus tridentatus* (Japanese horseshoe crab) |
| tr\|B7PHW6\|B7PHW6_IXOSC | - + | | | | | | | 20.79 | 1 | 1 | 1 | - | 2.86 | Acyl-CoA synthetase, putative. *Ixodes scapularis (Black- legged tick) (Deer tick)* |
| tr\|C3RRY0\|C3RRY0_9ARAC | + - | 33.69 | 1 | 1 | 1 | - | 9.78 | | | | | | | Histone H3. *Nephilengys sp. FAPDNA032* |
| tr\|T1KGY2\|T1KGY2_TETUR | + - | 53.74 | 2 | 1 | 1 | - | 6.92 | | | | | | | Histone H2A. OS= Tetranychus urticae |
| tr\|B2XY36\|B2XY36_LATHE | + + | 89.3 | 3 | 2 | 1 | - | 8.63 | 118.9 | 4 | 2 | 2 | - | 8.63 | Actin. *Latrodectus hesperus (Western black widow spider)* |
| tr\|A0A1S6G9Z6\|A0A1S6G9Z6_9ARAC | + - | 33.69 | 1 | 1 | 1 | - | 8.49 | | | | | | | Histone H3. *Pahorinae sp. New Zealand CG226* |
| tr\|Q9U900\|Q9U900_TACTR | + + | 93.39 | 8 | 3 | 3 | - | 13.86 | 98.6 | 15 | 2 | 1 | - | 13.86 | Pentaxin. OS= *Tachypleus tridentatus (Japanese horseshoe crab)* |
| tr\|A0A097CK67\|A0A097CK67_HAEFA | + + | 89.3 | 3 | 2 | 1 | - | 7.18 | 118.9 | 4 | 2 | 2 | - | 7.18 | Actin OS=Haemaphysalis flava PE=2 SV=1 |

| | | | | | | | | | | | | | | | | |
|---|---|---|---|---|---|---|---|---|---|---|---|---|---|---|---|---|
| tr\|A0A1E1WWZ1\|A0A1E1WWZ1_9ACAR | + - | 53.74 | 2 | 1 | 1 | - | 7.03 | | | | | | | Histone H2A. *Amblyoma aureolatum* . |
| tr\|A0A023FKT1\|A0A023FKT1_9ACAR | + + | 89.3 | 3 | 2 | 1 | - | 7.18 | 118.9 | 4 | 2 | 2 | - | 7.18 | Putative actin. *Amblyoma cajennense (Cayenne tick)* |
| tr\|A0A1E1WWC5\|A0A1E1WWC5_TITOB | - + | | | | | | | 106.96 | 5 | 3 | 1 | - | 5.13 | Putative hemocyanin subunit. *Tityus obscurus (Amazonian scorpion) (Tityus cambridgei)* |
| tr\|Q6XJD4\|Q6XJD4_9ARAC | + - | 33.69 | 1 | 1 | 1 | - | 8.26 | | | | | | | Histone H3 (Fragment) OS=Enoplognatha caricis GN=H3A PE=3 SV=1 |
| tr\|A0A131YIH1\|A0A131YIH1_RHIAP | + - | 29.19 | 2 | 1 | 1 | - | 7.52 | | | | | | | Tick transposon. *Rhipicephalus appendiculatus (Brown ear tick)* |
| tr\|L7M669\|L7M669_9ACAR | + + | 53.74 | 2 | 1 | 1 | - | 6.47 | 74.19 | 3 | 2 | 2 | - | 30.22 | Histone H2A OS=Rhipicephalus pulchellus PE=2 SV=1 |
| tr\|A0A061QLG5\|A0A061QLG5_CUPSA | + + | 89.3 | 3 | 2 | 1 | - | 7.18 | 118.9 | 4 | 2 | 2 | - | 7.18 | Putative actin. *Cupiennius salei (American wandering spider)* |

| | | | | | | | | | | | | | | |
|---|---|---|---|---|---|---|---|---|---|---|---|---|---|---|
| tr\|A0A293MXJ2\|A0A293MXJ2_ORNER | + + | 53.74 | 2 | 1 | 1 | - | 7.26 | 79.56 | 3 | 2 | 2 | - | 33.87 | Histone H2A. *Ornithodoros erraticus (European soft tick) (Alectorobius erraticus)* |
| tr\|A0A023FFC5\|A0A023FFC5_9ACAR | + + | 53.74 | 2 | 1 | 1 | - | 6.47 | 74.19 | 3 | 2 | 2 | - | 30.22 | Histone H2A. *Amblyomma cajennense (Cayenne tick)* |
| tr\|A1X1V5\|A1X1V5_CARRO | + + | 164.07 | 12 | 7 | 5 | - | 15.87 | 262.52 | 25 | 15 | 10 | - | 26.76 | Hemocyanin subunit IV OS=Carcinoscorpius rotundicauda PE=2 SV=1 |
| tr\|A0A076L2M6\|A0A076L2M6_NEPPI | + - | 81.22 | 3 | 2 | 1 | - | 8.06 | | | | | | | BLTX512. *Nephila pilipes (Giant wood spider) (Nephila maculata)* |
| tr\|A0A1E1X349\|A0A1E1X349_9ACAR | + + | 89.3 | 3 | 2 | 1 | - | 7.18 | 118.9 | 4 | 2 | 2 | - | 7.18 | Putative actin related protein 1. *Amblyomma aureolatum* |
| tr\|B6CEN8\|B6CEN8_9ARAC | + - | 33.69 | 1 | 1 | 1 | - | 8.26 | | | | | | | Histone H3 (Fragment) OS=Thomisus sp. B SPB-2007 PE=3 SV=1 |
| tr\|A0A224YY42\|A0A224YY42_9ACAR | + + | 53.74 | 2 | 1 | 1 | - | 6.47 | 74.19 | 3 | 2 | 2 | - | 30.22 | Histone H2A. *Rhipicephalus zambeziensis* |
| tr\|G8YZR0\|G8YZR0_LIMPO | + + | 139.93 | 7 | 5 | 2 | - | 10.67 | 186.69 | 14 | 8 | 4 | - | 18.79 | Hemocyanin subunit IIIb OS=Limulus |

| | | | | | | | | | | | | | | |
|---|---|---|---|---|---|---|---|---|---|---|---|---|---|---|
| | | | | | | | | | | | | | | polyphemus GN=HcIIIb PE=2 SV=1 |
| tr\|A0A1W6QBC5\|A0A1W6QBC5_9ARAC | + - | 33.69 | 1 | 1 | 1 | - | 9.09 | | | | | | | Histone 3. *Sinocoelotes pseudoyunnanensis* |
| tr\|A0A125RQ61\|A0A125RQ61_9ARAC | + - | 33.69 | 1 | 1 | 1 | - | 7.83 | | | | | | | Histone H3. *Araneidae sp. D MG-2015* |
| tr\|A0A1S6G970\|A0A1S6G970_9ARAC | + - | 33.69 | 1 | 1 | 1 | - | 8.49 | | | | | | | Histone H3. *Ferrieria echinate* |
| tr\|Q9TWE5\|Q9TWE5_CARRO | - + | | | | | | | 192.7 | 10 | 8 | 2 | - | 18.68 | Hemocyanin subunit HR6 OS=Carcinoscorpius rotundicauda PE=1 SV=1 |
| tr\|Q9U8Y8\|Q9U8Y8_TACTR | + + | 93.39 | 8 | 3 | 3 | - | 13.86 | 98.6 | 15 | 2 | 1 | - | 13.86 | Pentaxin. OS= *Tachypleus tridentatus* (Japanese horseshoe crab) |
| tr\|A0A1I9J788\|A0A1I9J788_9ARAC | + - | 33.69 | 1 | 1 | 1 | - | 8.26 | | | | | | | Histone H3. *Physoglenes sp. LB-2014a* |
| tr\|G3MLM7\|G3MLM7_9ACAR | + + | 89.3 | 3 | 2 | 1 | - | 7.18 | 118.9 | 4 | 2 | 2 | - | 7.18 | Uncharacterized protein OS=Amblyomma maculatum PE=2 SV=1 |
| tr\|B7SP61\|B7SP61_DERVA | + - | 53.74 | 2 | 1 | 1 | - | 7.03 | | | | | | | Histone H2A OS=Dermacentor variabilis PE=2 SV=1 |
| tr\|A0A2I4K5F2\|A0A2I4K5F2_9ACAR | + - | 53.74 | 2 | 1 | 1 | - | 7.03 | | | | | | | Histone H2A. *Rhipicephalus annulatus* |

| | | | | | | | | | | | | | | |
|---|---|---|---|---|---|---|---|---|---|---|---|---|---|---|
| tr\|A0A1E1WZ70\|A0A1E1WZ70_9ACAR | + + | 53.74 | 2 | 1 | 1 | - | 6.47 | 74.19 | 3 | 2 | 2 | - | 30.22 | Histone H2A. *Amblyomma aureolatum* |
| tr\|A1X1V7\|A1X1V7_CARRO | + + | 46.83 | 2 | 1 | 1 | - | 1.58 | 140.45 | 8 | 5 | 3 | - | 11.57 | Hemocyanin subunit VI OS=Carcinoscorpius rotundicauda PE=2 SV=1 |
| tr\|Q9U8Z6\|Q9U8Z6_TACTR | + + | 93.39 | 8 | 3 | 3 | - | 13.86 | 98.6 | 15 | 2 | 1 | - | 13.86 | Pentaxin. *Tachypleus tridentatus (Japanese horseshoe crab)* |
| tr\|A0A2H4IW89\|A0A2H4IW89_9ARAC | + - | 33.69 | 1 | 1 | 1 | - | 8.26 | | | | | | | Histone H3. *Tasmanoonops sp. CASENT9035003* |
| tr\|A1X1V4\|A1X1V4_CARRO | + + | 135.2 | 6 | 4 | 1 | - | 7.96 | 170.76 | 16 | 6 | 2 | - | 12.42 | Hemocyanin subunit IIIb OS=Carcinoscorpius rotundicauda PE=2 SV=1 |
| tr\|A0A293N6F4\|A0A293N6F4_ORNER | + - | 53.74 | 2 | 1 | 1 | - | 7.03 | | | | | | | Histone H2A. *Ornithodoros erraticus (European soft tick) (Alectorobius erraticus)* |
| tr\|A0A0B5H278\|A0A0B5H278_9ARAC | + - | 33.69 | 1 | 1 | 1 | - | 10.34 | | | | | | | Histone H3. *Cymbacha sp. JR-2014* |
| tr\|Q9U8Y7\|Q9U8Y7_TACTR | + + | 93.39 | 8 | 3 | 3 | - | 13.86 | 98.6 | 15 | 2 | 1 | - | 13.86 | Pentaxin. *Tachypleus tridentatus* |

| | | | | | | | | | | | | | | | |
|---|---|---|---|---|---|---|---|---|---|---|---|---|---|---|---|
| | | | | | | | | | | | | | | | *(Japanese horseshoe crab)* |
| tr\|S5ZGI8\|S5ZGI8_9ARAC | + - | 33.69 | 1 | 1 | 1 | - | 8.26 | | | | | | | | Histone H3 (Fragment) OS=Brachythele sp. CRBAMM000033 GN=H3 PE=3 SV=1 |
| tr\|Q9U8Z4\|Q9U8Z4_TACTR | + + | 93.39 | 8 | 3 | 3 | - | 13.86 | 98.6 | 15 | 2 | 1 | - | 13.86 | | Pentaxin. *Tachypleus tridentatus (Japanese horseshoe crab)* |
| tr\|Q95ZH4\|Q95ZH4_CUPSA | - + | | | | | | | 110.03 | 5 | 3 | 1 | - | 4.15 | | Hemocyanin subunit 6 OS=Cupiennius salei GN=hc-6 PE=2 SV=1 |
| tr\|A0A023FHJ9\|A0A023FHJ9_9ACAR | + - | 53.74 | 2 | 1 | 1 | - | 7.03 | | | | | | | | Histone H2A. *Amblyomma cajennense (Cayenne tick)* |
| tr\|D9IFL5\|D9IFL5_9ACAR | + + | 89.3 | 3 | 2 | 1 | - | 7.18 | 118.9 | 4 | 2 | 2 | - | 7.18 | | Actin OS=Hyalomma asiaticum PE=2 SV=1 |
| tr\|T1K0X9\|T1K0X9_TETUR | + + | 89.3 | 3 | 2 | 1 | - | 7.18 | 118.9 | 4 | 2 | 2 | - | 7.18 | | Uncharacterized protein OS=Tetranychus urticae PE=3 SV=1 |
| tr\|A0A1E1WZS9\|A0A1E1WZS9_9ACAR | + + | 53.74 | 2 | 1 | 1 | - | 7.26 | 79.56 | 3 | 2 | 2 | - | 33.87 | | Histone H2A. *Amblyomma* |

| | | | | | | | | | | | | | | |
|---|---|---|---|---|---|---|---|---|---|---|---|---|---|---|
| | | | | | | | | | | | | | | *aureolatum* |
| tr\|A0A076L049\|A0A076L049_NEPPI | + + | 89.3 | 3 | 2 | 1 | - | 8.36 | 118.9 | 4 | 2 | 2 | - | 8.36 | BLTX441. *Nephila pilipes (Giant wood spider) (Nephila maculata)* |
| tr\|G3MK37\|G3MK37_9ACAR | + + | 89.3 | 3 | 2 | 1 | - | 7.18 | 118.9 | 4 | 2 | 2 | - | 7.18 | Putative uncharacterized protein OS=Amblyomma maculatum PE=2 SV=1 |
| tr\|A0A147BCH6\|A0A147BCH6_IXORI | - + | | | | | | | 22.44 | 1 | 1 | 1 | - | 1.25 | Putative leucine-rich repeat protein. *Ixodes ricinus (Common tick) (Acarus ricinus)* |
| tr\|A0A1E1XK90\|A0A1E1XK90_9ACAR | - + | | | | | | | 31.59 | 1 | 1 | 1 | - | 1.82 | Putative chromatin remodelling complex wstf-iswi. *Amblyomma sculptum* |
| tr\|E5DRL7\|E5DRL7_9ARAC | + - | 33.69 | 1 | 1 | 1 | - | 8.26 | | | | | | | Histone H3 (fragment) OS=Patu sp. SYMP- 001-DR GN=H3 PE=3 SV=1 |
| tr\|C6H102\|C6H102_PANIM | - + | | | | | | | 107.08 | 5 | 3 | 1 | - | 4.27 | Hemocyanin subunit 5b. *Pandinus imperator (Emperor scorpion)* |

| | | | | | | | | | | | | | | |
|---|---|---|---|---|---|---|---|---|---|---|---|---|---|---|
| tr\|Q9U8Z8\|Q9U8Z8_TACTR | + + | 93.39 | 8 | 3 | 3 | - | 13.86 | 98.6 | 15 | 2 | 1 | - | 13.86 | Pentaxin. *Tachypleus tridentatus (Japanese horseshoe crab)* |
| tr\|A0A0S1LJE0\|A0A0S1LJE0_9ARAC | + - | 33.69 | 1 | 1 | 1 | - | 8.33 | | | | | | | Histone H3. *Anyphaena accentuata* |
| tr\|Q9U8Z2\|Q9U8Z2_TACTR | + + | 93.39 | 8 | 3 | 3 | - | 13.86 | 98.6 | 15 | 2 | 1 | - | 13.86 | Pentaxin. *Tachypleus tridentatus (Japanese horseshoe crab)* |
| tr\|A0A023FIL9\|A0A023FIL9_9ACAR | + + | 89.3 | 3 | 2 | 1 | - | 7.18 | 118.9 | 4 | 2 | 2 | - | 7.18 | Putative actin OS=Amblyomma cajennense PE=2 SV=1 |
| tr\|A0A076KZN4\|A0A076KZN4_NEPPI | + + | 89.3 | 3 | 2 | 1 | - | 8.28 | 118.9 | 4 | 2 | 2 | - | 8.28 | BLTX465 (actin). *Nephila pilipes (Giant wood spider) (Nephila maculata)* |
| tr\|T1KE54\|T1KE54_TETUR | + + | 89.3 | 3 | 2 | 1 | - | 7.18 | 118.9 | 4 | 2 | 2 | - | 7.18 | Uncharacterized protein. *Tetranychus urticae (Two-spotted spider mite)* |
| tr\|A0A1V0CHF6\|A0A1V0CHF6_9ARAC | + - | 33.69 | 1 | 1 | 1 | - | 8.26 | | | | | | | Histone H3. *Nemesia sp. n. Minorca* |
| tr\|A1X1V6\|A1X1V6_CARRO | + + | 34.24 | 2 | 1 | 1 | - | 1.1 | 221.66 | 28 | 15 | 13 | - | 26.18 | Hemocyanin subunit V. *Carcinoscorpius rotundicauda (Mangrove horseshoe crab)* |

| | | | | | | | | | | | | | | |
|---|---|---|---|---|---|---|---|---|---|---|---|---|---|---|
| | | | | | | | | | | | | | | *(Limulus rotundicauda)* |
| tr\|A0A076KTV1\|A0A076KTV1_NEPPI | + + | 89.3 | 3 | 2 | 1 | - | 8.33 | 118.9 | 4 | 2 | 2 | - | 8.33 | BLTX369 (actin family). *Nephila pilipes (Giant wood spider) (Nephila maculata)* |
| tr\|M4HY71\|M4HY71_9ARAC | + - | 33.69 | 1 | 1 | 1 | - | 8.26 | | | | | | | Histone H3 (fragment) OS=Pholcus chappuisi GN=H3 PE=3 SV=1 |
| tr\|L7M671\|L7M671_9ACAR | + + | 53.74 | 2 | 1 | 1 | - | 7.26 | 79.56 | 3 | 2 | 2 | - | 33.87 | Histone H2A OS=Rhipicephalus pulchellus PE=2 SV=1 |
| tr\|T1KE55\|T1KE55_TETUR | + + | 89.3 | 3 | 2 | 1 | - | 7.18 | 118.9 | 4 | 2 | 2 | - | 7.18 | Uncharacterized protein. *Tetranychus urticae (Two-spotted spider mite)* |
| tr\|A0A0C9S0H3\|A0A0C9S0H3_AMBAM | + + | 53.74 | 2 | 1 | 1 | - | 7.26 | 79.56 | 3 | 2 | 2 | - | 33.87 | Histone H2A. *Amblyomma americanum (Lone star tick)* |
| tr\|A0A131YWD3\|A0A131YWD3_RHIAP | + - | 53.74 | 2 | 1 | 1 | - | 7.03 | | | | | | | Histone H2A. *Rhipicephalus appendiculatus (Brown ear tick)* |
| tr\|A1X1V2\|A1X1V2_CARRO | - + | | | | | | | 191.48 | 13 | 8 | 6 | - | 16.85 | Hemocyanin subunit II OS=Carcinoscorpius rotundicauda |

| | | | | | | | | | | | | | | |
|---|---|---|---|---|---|---|---|---|---|---|---|---|---|---|
| | | | | | | | | | | | | | | PE=2<br>SV=1 |
| tr\|A0A0K1DCW9\|A0A0K1DCW9_9ARAC | + - | 33.69 | 1 | 1 | 1 | - | 8.33 | | | | | | | Histone H3. *Horribates bantai* |
| tr\|A0A293LUJ7\|A0A293LUJ7_ORNER | + - | 26.66 | 2 | 1 | 1 | - | 0.99 | | | | | | | DNA polymerase. *Ornithodoros erraticus (European soft tick) (Alectorobius erraticus)* |
| tr\|A0A1E1XK53\|A0A1E1XK53_9ACAR | - + | | | | | | | 31.59 | 1 | 1 | 1 | - | 2.24 | Putative chromatin remodeling complex wstf-iswi. *Amblyomma sculptum* |
| tr\|E7D193\|E7D193_LATHE | + - | 89.3 | 3 | 2 | 1 | - | 8.16 | | | | | | | Actin 5C. *Latrodectus hesperus (Western black widow spider)* |
| tr\|A1X1V3\|A1X1V3_CARRO | + + | 135.3 | 14 | 7 | 7 | - | 15.85 | 204.69 | 31 | 14 | 12 | - | 24.25 | Hemocyanin subunit IIIa OS=Carcinoscorpius rotundicauda PE=2 SV=1 |
| sp\|P15566\|COAG_TACGI | + + | 242.5 | 39 | 15 | 15 | - | 83.43 | 289.33 | 46 | 16 | 16 | - | 77.71 | Coagulogen. *Tachypleus gigas (Southeast Asian horseshoe crab)* |
| tr\|I3XK82\|I3XK82_TACTR | + + | 89.3 | 3 | 2 | 1 | - | 7.18 | 118.9 | 4 | 2 | 2 | - | 7.18 | Beta-actin OS=Tachypleus tridentatus PE=2 SV=1 |

| | | | | | | | | | | | | | | |
|---|---|---|---|---|---|---|---|---|---|---|---|---|---|---|
| tr\|G3MNR4\|G3MNR4_9ACAR | + - | 53.74 | 2 | 1 | 1 | - | 7.03 | | | | | | | Histone H2A OS=Amblyomma maculatum PE=2 SV=1 |
| tr\|E5DRJ7\|E5DRJ7_9ARAC | + - | 33.69 | 1 | 1 | 1 | - | 8.26 | | | | | | | Histone H3 (fragment). OS=Mysmeninae sp. MYSM-019-MAD GN=H3 PE=3 SV=1 |
| tr\|Q7M490\|Q7M490_LIMPO | + - | 34.24 | 2 | 1 | 1 | - | 29.17 | | | | | | | Hemocyanin subunit V. *Limulus polyphemus (Atlantic horseshoe crab)* |
| tr\|Q9U8Z7\|Q9U8Z7_TACTR | + + | 93.39 | 8 | 3 | 3 | - | 13.86 | 98.6 | 15 | 2 | 1 | - | 13.86 | Pentaxin. *Tachypleus tridentatus (Japanese horseshoe crab)* |
| tr\|Q95ZH2\|Q95ZH2_CUPSA | - + | | | | | | | 110.03 | 5 | 3 | 1 | - | 4.15 | Hemocyanin subunit 6" OS=Cupiennius salei GN=hc-6" PE=2 SV=1 |
| tr\|A0A023GH79\|A0A023GH79_9ACAR | + - | 53.74 | 2 | 1 | 1 | - | 7.03 | | | | | | | Histone H2A. *Amblyomma triste* |
| tr\|B8YDK9\|B8YDK9_ARGAU | + - | 33.69 | 1 | 1 | 1 | - | 8.26 | | | | | | | Histone H3 (fragment) OS=Argiope aurantia GN=H3 PE=3 SV=1 |
| tr\|A0A1V0CHL0\|A0A1V0CHL0_9ARAC | + - | 33.69 | 1 | 1 | 1 | - | 8.26 | | | | | | | Histone H3. *Nemesia sp. CRBAMM001004* |

| | | | | | | | | | | | | | | |
|---|---|---|---|---|---|---|---|---|---|---|---|---|---|---|
| tr\|A0A087UR67\|A0A087UR67_9ARAC | - + | | | | | | | 106.01 | 5 | 3 | 1 | - | 5.37 | Hemocyanin A chain. *Stegodyphus mimosarum (African social velvet spider)* |
| tr\|T1Z0E6\|T1Z0E6_9ARAC | + - | 33.69 | 1 | 1 | 1 | - | 9 | | | | | | | Histone H3 (Fragment) OS=Phonognatha graeffei GN=H3 PE=3 SV=1 |
| tr\|A0A2I4K566\|A0A2I4K566_9ACAR | + + | 53.74 | 2 | 1 | 1 | - | 7.26 | 79.56 | 3 | 2 | 2 | - | 33.87 | Histone H2A. *Rhipicephalus annulatus* |
| tr\|Q95ZH3\|Q95ZH3_CUPSA | - + | | | | | | | 110.03 | 5 | 3 | 1 | - | 4.15 | Hemocyanin subunit 6' OS=Cupiennius salei GN=hc-6' PE=2 SV=1 |
| A5EVP6\|PYRB_DICNV | ++ | | | | | | | | | | | | | Aspartate carbamoyltransferase catalytic subunit (Dichelobacter nodosus VCS1703A) |
| A5WH78\|PROA_PSYWF | ++ | | | | | | | | | | | | | Gamma-glutamyl phosphate reductase (Psychrobacter sp. PRwf-1) |

| | | | | | | | | | | | | | | |
|---|---|---|---|---|---|---|---|---|---|---|---|---|---|---|
| Q4FUZ5\|PROA_PSYA2 | ++ | | | | | | | | | | | | | Gamma-glutamyl phosphate reductase (Psychrobacter arcticus ) |
